# Linking Microstructural Integrity and Motor Cortex Excitability in Multiple Sclerosis

**DOI:** 10.3389/fimmu.2021.748357

**Published:** 2021-10-12

**Authors:** Angela Radetz, Kalina Mladenova, Dumitru Ciolac, Gabriel Gonzalez-Escamilla, Vinzenz Fleischer, Erik Ellwardt, Julia Krämer, Stefan Bittner, Sven G. Meuth, Muthuraman Muthuraman, Sergiu Groppa

**Affiliations:** ^1^ Neuroimaging and Neurostimulation, Department of Neurology, Focus Program Translational Neuroscience (FTN), Rhine-Main Neuroscience Network (rmn2), University Medical Center of the Johannes Gutenberg-University Mainz, Mainz, Germany; ^2^ Laboratory of Neurobiology and Medical Genetics, Nicolae Testemițanu State University of Medicine and Pharmacy, Chişinău, Moldova; ^3^ Department of Neurology, Institute of Emergency Medicine, Chişinău, Moldova; ^4^ Department of Neurology, Institute of Translational Neurology, University Hospital Münster, Münster, Germany; ^5^ Department of Neurology, University Hospital Düsseldorf, Düsseldorf, Germany

**Keywords:** multiple sclerosis, neurite orientation dispersion and density imaging, NODDI, excitability, motor threshold, tract-based spatial statistics

## Abstract

Motor skills are frequently impaired in multiple sclerosis (MS) patients following grey and white matter damage with cortical excitability abnormalities. We applied advanced diffusion imaging with 3T magnetic resonance tomography for neurite orientation dispersion and density imaging (NODDI), as well as diffusion tensor imaging (DTI) in 50 MS patients and 49 age-matched healthy controls to quantify microstructural integrity of the motor system. To assess excitability, we determined resting motor thresholds using non-invasive transcranial magnetic stimulation. As measures of cognitive-motor performance, we conducted neuropsychological assessments including the Nine-Hole Peg Test, Trail Making Test part A and B (TMT-A and TMT-B) and the Symbol Digit Modalities Test (SDMT). Patients were evaluated clinically including assessments with the Expanded Disability Status Scale. A hierarchical regression model revealed that lower neurite density index (NDI) in primary motor cortex, suggestive for axonal loss in the grey matter, predicted higher motor thresholds, i.e. reduced excitability in MS patients (*p* = .009, adjusted r² = 0.117). Furthermore, lower NDI was indicative of decreased cognitive-motor performance (*p* = .007, adjusted r² = .142 for TMT-A; *p* = .009, adjusted r² = .129 for TMT-B; *p* = .006, adjusted r² = .142 for SDMT). Motor WM tracts of patients were characterized by overlapping clusters of lowered NDI (*p* <.05, Cohen’s d = 0.367) and DTI-based fractional anisotropy (FA) (*p* <.05, Cohen’s d = 0.300), with NDI exclusively detecting a higher amount of abnormally appearing voxels. Further, orientation dispersion index of motor tracts was increased in patients compared to controls, suggesting a decreased fiber coherence (*p* <.05, Cohen’s d = 0.232). This study establishes a link between microstructural characteristics and excitability of neural tissue, as well as cognitive-motor performance in multiple sclerosis. We further demonstrate that the NODDI parameters neurite density index and orientation dispersion index detect a larger amount of abnormally appearing voxels in patients compared to healthy controls, as opposed to the classical DTI parameter FA. Our work outlines the potential for microstructure imaging using advanced biophysical models to forecast excitability alterations in neuroinflammation.

## Introduction

Impaired motor functions caused by both grey (GM) and white matter (WM) pathology are characteristic of multiple sclerosis (MS), a neurodegenerative inflammatory disorder of the central nervous system (1–3). Both in animal models of MS and in patients, cortical neurons show abnormal excitability levels (4, 5). Excitability of neural tissue in motor brain regions can be measured *in vivo* by determining the resting motor threshold with transcranial magnetic stimulation (TMS), which is frequently observed to be increased in MS patients (6, 7). Although the driving mechanisms of altered cortical excitability are still unclear, cortical neuronal or axonal loss or altered (re-) myelination processes could play an important role (7, 8). By applying biophysical models on diffusion-weighted images (DWI), microstructural characteristics of the underlying tissue can be determined. Conventional DWI parameters that are derived from fitted diffusion tensors (diffusion tensor imaging, DTI) include fractional anisotropy (FA), mean (MD), radial (RD) and axial diffusivity (AD). Increased MD, RD and AD along with decreased FA has been observed in patients with MS, which can be explained by a loss or an altered organization of structural barriers, leading to more isotropic diffusion profiles (9–12). As FA incorporates diffusivity in all three spatial dimensions and is a frequently reported measure in MS research, we here focused on this DTI parameter. Whereas FA has been particularly applied to assess white matter (WM) pathology, this measure is not specific to the type of neuroinflammation- or neurodegeneration-driven tissue damage (13–15). Further, FA does not distinguish restricted and hindered diffusion and is thereby biased in areas with high neurite orientation dispersion as the GM or regions of crossing fibers (16, 17). Advanced biophysical models such as the neurite orientation dispersion and density imaging (NODDI) model, which can be applied to both the GM and WM, allow a more realistic in-vivo depiction of microstructure including estimations of neurite density and dispersion (17). This model is therefore an interesting candidate for early predictions of microstructural changes and their neurological and behavioral consequences in the diseased brain. In each voxel a neurite density (NDI) and orientation dispersion index (ODI) is computed from restricted diffusion, whereas an isotropic volume fraction (IVF) reflects cerebrospinal fluid and edema (17). The NODDI model was validated repeatedly including a mouse histology study and a cross-modality study of the human cortical GM (18, 19). Here, we aimed at carrying forward the potential of the model to evaluate the microstructural integrity in the GM of primary motor cortex and address the question, whether lower excitability is related to NODDI parameters and FA.

A previous longitudinal investigation involving patients with mild traumatic brain injury (20) showed that NODDI appeared as a more sensitive model than DTI for microstructural changes and the relation to neuropsychological performance. We hence hypothesized that NODDI measures used for quantification of motor microstructural integrity are predictive for motor and cognitive function in MS patients, while we also considered FA in the statistical model. To that end, we used neuropsychological tests that are commonly reported in MS research and contain both a motor and cognitive component.

As MS pathology occurs in the GM and WM of the motor system, we further aimed at comparing microstructural integrity in motor WM tracts of MS patients and HC using tract-based spatial statistics (TBSS) (21). Importantly, we investigated if NODDI values are more sensitive in detecting differences in microstructural integrity in MS patients versus HC in contrast to the classical FA. As FA increases might reflect either NDI increases, ODI decreases or a combination of both (17), we tracked contributions of these signals to FA and evaluated if the NODDI model captures additional pathological alterations due to MS pathology.

We first assessed (1a) the predictive value of motor cortex microstructure for motor excitability and (1b) cognitive-motor performance in our participants. Next, we evaluated (2) pathological microstructure alterations in motor WM tracts of MS patients and HC using NODDI in comparison to the classical FA. We hereby examined voxel-wise intersecting group differences to disentangle potential contributions of NODDI metrics to FA.

## Materials and Methods

### Ethical Approval of the Study

The study was approved by the local Ethics Committee of the State Medical Association of Rhineland-Palatinate.

### Participants

Fifty patients with relapsing-remitting MS (RRMS) (31 female, mean age = 35.3 years, SD = 11.1), diagnosed according to the revised McDonald criteria (22), and 49 HC (25 female, mean age = 31.5 years, SD = 9.1) participated in our study. HC and MS were all right-handed and did not significantly differ in age or gender distribution (both *p* >.05). Clinical data of patients, Nine Hole Peg Test (9HPT), Trail Making Test part A (TMT-A) and part B (TMT-B) and Symbol Digit Modalities Test (SDMT) raw scores (23) and an overview of ongoing therapies at the time of MRI acquisition are depicted in **Table 1**. Participants were excluded from the study in case of pregnancy or relapses or systemic therapy with steroids within the month before MRI. They were also excluded in case of contraindication to TMS, i.e. previous or current susceptibility to epileptic seizures or metal implants in proximity to the cranium. Expanded Disability Status Scale (EDSS) scores (3) of MS patients were determined by trained and certified neurologists. All participants gave written informed consent before participation and were informed about the study content in oral and written form.

**Table 1:** Overview of patients’ clinical characteristics.

Disease duration in months, mean ± SD	50 ± 59
EDSS, median (interquartile range)	1 (1.25)
Total lesion volume in ml, mean ± SD	3.88 ± 5.98
9HPT raw score, mean ± SD	67.47 ± 13.48
TMT-A raw score, mean ± SD	25.40 ± 11.82
TMT-B raw score, mean ± SD	52.50 ± 28.04
SDMT raw score, mean ± SD	53.32 ± 10.50
Disease modifying therapies	
Dimethyl fumarate	14
Beta interferons	10
Glatiramer acetate	5
Natalizumab	5
Fingolimod	3
Alemtuzumab	2
Daclizumab	2
Teriflunomide	2
Cladribine	1
Ocrelizumab	1
No therapy	5

EDSS, Expanded Disability Status Scale; 9HPT, Nine Hole Peg Test; TMT-A, Trail Making Test part A; TMT-B, Trail Making Test part B; SDMT, Symbol Digit Modalities Test; SD, standard deviation.

### MRI

Scanning was performed in a 3T Siemens TrioTim MRI scanner with a 32-channel head coil (Siemens Healthcare, Erlangen, Germany). A 3D T1-weighted magnetization prepared rapid gradient echo (MP-RAGE) sequence (echo time [TE] = 2.52 ms, repetition time [TR] = 1900 ms, inversion time [TI] = 900 ms, flip angle = 9°, matrix size = 256 x 256, field of view [FOV] = 256 x 256 mm², slice thickness = 1 mm, voxel size = 1 x 1 x 1 mm³) and a sagittal 3D turbo spin-echo fluid attenuated inversion recovery (FLAIR) sequence (TE = 388 ms, TR = 5000 ms, TI = 1800 ms, flip angle = 120°, matrix size = 256 x 258, FOV = 256 x 256 mm², slice thickness = 1 mm, voxel size = 0.5 x 0.5 x 1 mm³) were acquired. Further, multi-shell DWI data were obtained (TE = 111 ms, TR = 10800 ms, flip angle = 90°, matrix size = 128 x 128, FOV = 2304 x 2304 mm², slice thickness = 2 mm, voxel size = 2 x 2 x 2 mm³), with three non-zero *b*-values each measured in thirty unique directions (*b* = 900 s/mm², *b* = 1800 s/mm², *b* = 2700 s/mm²) in the anterior-posterior direction. Six non diffusion-weighted volumes were acquired before each change in *b*-value in the anterior-posterior direction. Finally, one further non diffusion-weighted volume was acquired in anterior-posterior, and one in posterior-anterior direction.

### Transcranial Magnetic Stimulation and Recordings of Motor Evoked Potentials

Electromyography (EMG) electrodes were placed over the right first dorsal interosseous (FDI) and abductor pollicis brevis (APB). EMG signals including the motor evoked potentials (MEP) were 1000-fold amplified with a D440 amplifier from Digitimer (Fort Lauderdale, USA). Using a CED 1401 laboratory interface, signals were digitized with a sampling rate of 5 kHz (Cambridge Electronic Design, Cambridge, UK). TMS was applied using a Rapid² Stimulator with a figure-eight coil in biphasic pulse configuration (Magstim^®^, Whitland, UK). The TMS coil was placed over the hand area of left primary motor cortex (M1) approximately in parallel to the central sulcus, i.e. 45-55° relative to the mid-sagittal line (24). After each suprathreshold pulse consistently evoked an MEP in the right FDI and APB, the individual resting motor threshold was determined. To that end, pulse intensity was lowered until MEPs with peak-to-peak amplitudes of 50 *μV* were evoked for two out of four pulses at rest (25).

### Neuropsychological Assessment

In the MS group, 31 patients underwent neuropsychological cognitive assessment of 9HPT, and 44 patients of TMT-A, TMT-B and SDMT (time interval between MRI and neuropsychological testing was mean = 3.8 months, SD = 3.1 months). The 9HPT is a frequently used measure of manual dexterity that is often impaired in MS (26). Participants are asked to place and remove pegs into holes on a board as quickly as possible. We used 9HPT scores of the dominant (right) hand as we accordingly investigated the left-hemispheric M1 cortex. In the TMT, patients are instructed to connect a set of numbers and letters according to specific rules as fast as possible. Whereas we included the test due to the evaluation of motor speed, it also requires visual attention and task switching abilities. Lastly, we assessed the SDMT that can robustly capture cognitive impairment and is frequently in use for clinical evaluation of MS patients. Patients are asked to conduct a simple substitution task by pairing numbers with geometric figures. Here, SDMT was conducted in written form, such that motor speed was intrinsically assessed by the test as well. Average raw scores and standard deviations are reported in **Table 1**. We used z-scores of these variables by comparing the raw scores with test-specific normative data stratified for age and education.

### Data Preprocessing

**Figure 1:**
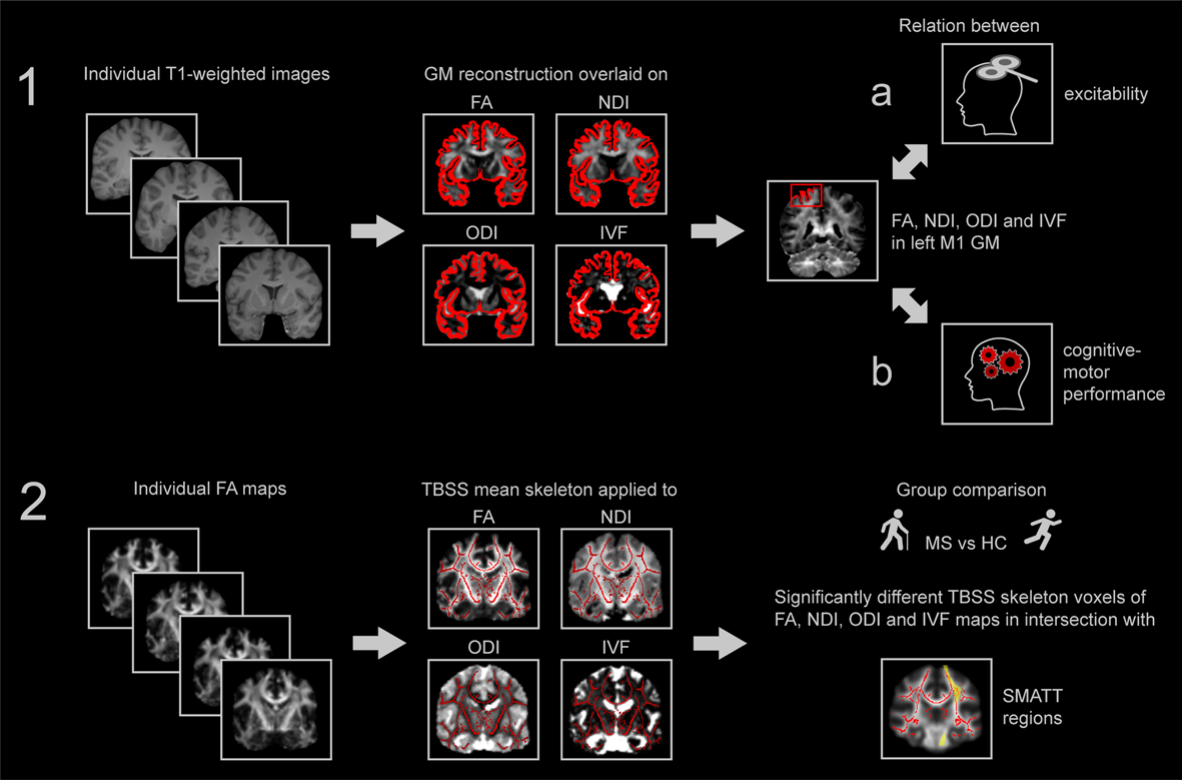
Study pipeline. (1) T1-weighted images were used for reconstruction of the GM that was individually coregistered to FA, NDI, ODI and IVF maps. Motor threshold as measure of excitability was regressed on average FA, NDI, ODI and IVF within the GM of left M1 (1a). Scores of cognitive-motor performance were similarly regressed on average FA, NDI, ODI and IVF within the GM of left M1 (1b). (2) Individual FA maps of all participants were used to compute the TBSS mean skeleton that was applied to FA, NDI, ODI and IVF for comparisons of MS and HC group. Percentage of intersecting significantly different voxels with regions of the sensorimotor area tract template (SMATT) (27) were determined for each diffusion parameter. FA, fractional anisotropy, NDI, neurite density index, ODI, orientation dispersion index, IVF, isotropic volume fraction, MS, multiple sclerosis, HC, healthy control, TBSS, tract-based spatial statistics, SMATT, sensorimotor area tract template, GM, grey matter, M1, primary motor cortex.

**Figure 1** provides an overview of the study pipeline.

### Brain Segmentation

Brain segmentation based on T1-weighted images was conducted using the cross-sectional processing stream of FreeSurfer version 5.3.0 (http://surfer.nmr.mgh.harvard.edu/). Here, removal of non-brain tissue, automated Talairach transformation, cortical and subcortical segmentation, intensity normalization, tessellation of the grey and white matter boundary and automated topology correction is included (28). We used the FreeSurfer-based GM segmentation of the cortical ribbon for further analyses.

### Preprocessing of DWI

DWI data were preprocessed using the diffusion toolbox of FSL version 5.0.9 (https://fsl.fmrib.ox.ac.uk/fsl/) by correcting susceptibility induced distortions with *topup*, in which we fed the seven non diffusion-weighted images acquired in the anterior-posterior and one in the posterior-anterior direction. We then used *eddy* for applying eddy current and motion artifact correction. Voxel-wise diffusion tensor fitting and computation of FA was performed using FSL’s *dtifit* only considering the inner shell (*b* = 900) and the non diffusion-weighted images.

### Application of the NODDI Model

Zhang and colleagues developed the NODDI model with the requirement of being sufficiently simple, while being complex enough for depiction of major characteristics of neurite morphology (17). A further aim was a clinically feasible acquisition time below 30 minutes. We fitted the model to the DWI using the Accelerated Microstructure Imaging *via* Convex Optimization (AMICO) algorithm resulting in NDI, ODI and IVF maps (29). Whereas IVF is characterized by free isotropic Gaussian diffusion, NDI and ODI are computed from restricted diffusion adapted from the orientation-dispersed cylinder model (17). We coregistered the first non diffusion-weighted image to the brainmask obtained with FreeSurfer using FSL’s FMRIB’s Linear Image Registration Tool (*FLIRT*). The transformation matrix was then applied to FA, NDI, ODI and IVF maps. This registration was performed for later masking with individual cortical ribbon masks obtained using FreeSurfer.

### Application of a Motor Atlas

The Human Motor Area Template (HMAT) contains six sensorimotor regions within each hemisphere (30). As the left M1 mask of the HMAT includes both GM and WM, we first also coregistered it to the brainmask in FreeSurfer space. This allowed us to compute average values of FA, NDI, ODI and IVF exclusively within the cortical ribbon of left M1 as reconstructed in FreeSurfer.

### Lesion Segmentation

We estimated lesion volumes with the lesion growth algorithm (31) implemented in the lesion segmentation toolbox (LST) version 2.0.15 (https://www.applied-statistics.de/lst.html). T2-FLAIR volumes were coregistered to the T1-weighted images and segmentation information used to compute lesion belief maps. After visual inspection, κ = 0.2 was chosen for thresholding of these maps. By growing lesions along hyperintensely appearing voxels in the T2-flair images, binary lesion maps were obtained. We linearly transformed T2-flair images to the brainmask obtained with FreeSurfer using FSL’s *FLIRT*. The transformation matrix was applied to transform lesion maps to FreeSurfer space. The percentage of lesion load in the left M1 mask was computed and used as covariate for regression analyses.

### Statistical Analyses

#### 1. Grey Matter Microstructural Integrity of the Motor System

Normality of data was assessed visually and using the Lilliefors test in *R* (version 3.6.3, *RStudio*). We then conducted group comparisons of motor threshold and FA, NDI, ODI and IVF in left M1 (**Supplementary Materials S2** and **S3**). Additionally, we tested homogeneity of variance between the groups using Levene’s test (**Supplementary Materials S2** and **S3**). Motor threshold and diffusion parameters were correlated using *R* package *corrplot* (**Supplementary Figure S1**). Here, significance (*p* <.05) after correction for multiple comparisons using false discovery rate (FDR) correction was indicated. These plots of correlation coefficients were further used for reports of the correlation between 9HPT, TMT-A, TMT-B, SDMT and diffusion parameters (**Supplementary Figure S2**).

For the main analysis, hierarchical backwards regression was conducted using *IBM SPSS Statistics for Windows*, Version 23 (IBM Corp., Armonk, NY) for the MS and HC group separately. As control variables, we entered gender and age in the model for prediction of the dependent variable motor threshold. Additionally, the percentage of lesion load in left M1 was considered as control variable for the MS model. All control variables were entered first in one block in the model. Variables of interest were FA, NDI, ODI and IVF averaged within the left M1 GM mask. Similarly, regression with backwards elimination was conducted for 9HPT, SDMT, TMT-A and TMT-B scores on FA, NDI, ODI and IVF in left M1 GM in MS patients. The order of removal of variables of interest was determined based on increase in correlation coefficients with the dependent variable (**Supplementary Figures S1** and **S2**), and predictors were only retained in case of *p* <.05 of the regression coefficient.

As a supplementary analysis, we also show coefficients of the correlation between diffusion parameters in a smaller hand area GM mask and motor threshold (**Supplementary Material S8**). Similarly, correlation coefficients are shown for a WM tract mask originating in left M1 as a control analysis. This mask was extracted from the Sensorimotor Area Tract Template (SMATT), where six sensorimotor cortical regions from the HMAT were used as starting points for probabilistic tractography, with waypoints positioned in the posterior limb of the internal capsule and the cerebral peduncle, while excluding transcallosal fibers (27). The correlation between diffusion parameters within the left M1 SMATT WM tract mask and motor threshold are depicted in **Supplementary Material S9**. For both analyses, we again used *R* package *corrplot.*

#### 2. White Matter Microstructural Integrity of the Motor System

First, TBSS was conducted with FA maps of both groups. This method allows voxel-wise group comparisons by non-linear registration and projection onto an alignment-invariant tract representation (21). As target for the nonlinear registration, the FMRIB58_FA_1mm standard space template as provided by FSL was used. The skeleton was thresholded to κ = 0.2 (21). The resulting skeleton was then also applied to NDI, ODI and IVF maps. Permutation test using the randomize tool was applied for statistical analyses (32). For each group comparison of FA, NDI, ODI and IVF between HC and MS, 500 permutations were carried out with the threshold-free cluster enhancement (TFCE) option to control for multiple comparisons. A threshold of *p* <.05 was used to define significance (33). As an effect size measure, we report Cohen’s d averaged over voxels that showed significant group differences. We overlaid all tract masks of the SMATT on the mean FA skeleton and computed the number of voxels in the skeleton for each SMATT region. We then extracted the number and percentage of voxels with significant group differences intersecting with SMATT tracts for FA, NDI, ODI and IVF maps, respectively. Lastly, we computed the percentage of overlapping group differences in all diffusion parameters per region, for all combinations of these parameters, as well as the averages over all regions.

## Results 

### 1. Grey Matter Microstructural Integrity of the Motor System

#### 1a. Microstructural Correlates of Motor Cortex Excitability

We first assessed group differences in average FA, NDI, ODI and IVF in the GM of left M1, and motor threshold, as well as their variances. Apart from a higher spread of NDI in the MS compared to the HC group, no significant differences were observed, and details are reported in **Supplementary Materials S2** and **S3**.

Correlation coefficients of the correlation between left M1 diffusion measures and motor threshold are visualized in **Supplementary Figure S1**.

**Figure 2:**
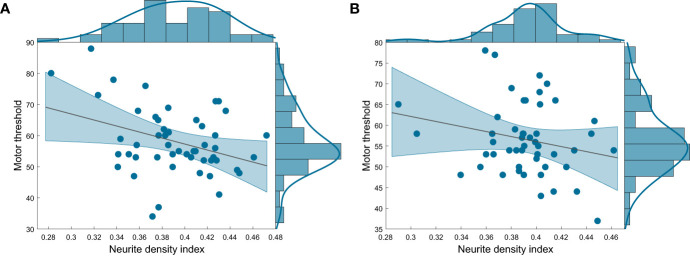
Scatterplot with regression line and density estimates of the regression of motor threshold on neurite density index in **(A)** patients with MS and **(B)** HC. HC, healthy controls, MS, multiple sclerosis.

In our main analysis, we first entered age and gender as potentially confounding variables into the hierarchical backward regression model for the prediction of motor threshold for MS and HC separately. Predicting motor threshold in the MS group, we additionally included lesion load in M1. As variables of interest, we considered FA, NDI, ODI and IVF in the GM of left M1, which served as the stimulation site for determination of the motor threshold. NDI in the GM of left M1 significantly predicted motor threshold in MS (F(1,48) = 7.493, *p* = .009) and explained 11.7% of its variance (for details of hierarchical regression analysis see **Supplementary Table S3**). **Figure 2A** depicts the relationship between the two variables and the respective density estimates, showing that lower neurite density in MS is linked to a higher motor threshold, i.e. lower excitability. No model was significant in the HC group, however there was similarly a trend for NDI predicting motor threshold (**Figure 2B** and **Supplementary Table S4**). By including NDI of both groups and a dummy variable encoding group membership for the prediction of motor threshold, we tested significance of group-specific slopes. The coefficient of the interaction term was not significant (*p* >.05), indicating that slopes were not significantly different between MS patients and HC.

To verify that the link between lower neurite density and higher motor threshold in MS patients was not dependent on the large size of the M1 mask, we also assessed the correlation coefficients between FA and NODDI values in a smaller hand area GM mask (**Supplementary Figure S3**). These were very similar to the main analysis that focused on the whole left M1 GM mask (**Supplementary Figure S1**). Furthermore, we assessed whether this link was specific to the GM or if there was also a correlation between FA, NDI, ODI and IVF within the SMATT WM tract originating in left M1 and motor threshold. FA and NODDI values within this WM tract did not correlate with motor threshold (**Supplementary Figure S4**).

#### 1b. Microstructural Correlates of Cognitive-Motor Performance

Correlation coefficients of the correlation between the diffusion parameters in left M1 and patient’s 9HPT, TMT-A, TMT-B and SDMT are depicted in **Supplementary Figure S2**.

**Figure 3:**
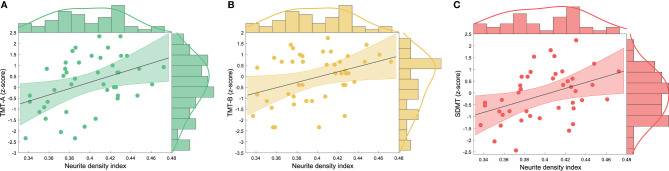
Scatterplot with regression line and density estimates of the regression of **(A)** TMT-A, **(B)** TMT-B and **(C)** SDMT z-scores on neurite density index in patients with MS. MS, multiple sclerosis, TMT-A, Trail Making Test part A, TMT-B, Trail Making Test part B, SDMT, Symbol Digit Modalities Test.

MS patient’s 9HPT scores were regressed on control variables age, gender and lesion load in M1 and variables of interest FA, NDI, ODI and IVF in left M1 GM. No variable of interest significantly predicted 9HPT scores (**Supplementary Table S5**). Equivalently, TMT-A, TMT-B and SDMT z-scores were regressed on control variables age, gender and lesion load in M1 and variables of interest FA, NDI, ODI and IVF in left M1 GM. NDI in the GM of left M1 was a significant predictor of TMT-A (F(1,42) = 8.102; *p* =.007; adjusted r² = .142), TMT-B (F(1,42) = 7.390; *p* =.009; adjusted r² = .129) and SDMT z-scores (F(1,42) = 8.260; *p* =.006; adjusted r² = .142) (for details of hierarchical regression analyses see **Supplementary Tables S5–S8**). Lower NDI predicted worse performance in TMT-A (**Figure 3A**), TMT-B (**Figure 3B**) and SDMT (**Figure 3C**).

### 2. White Matter Microstructural Integrity of the Motor System

**Figure 4:**
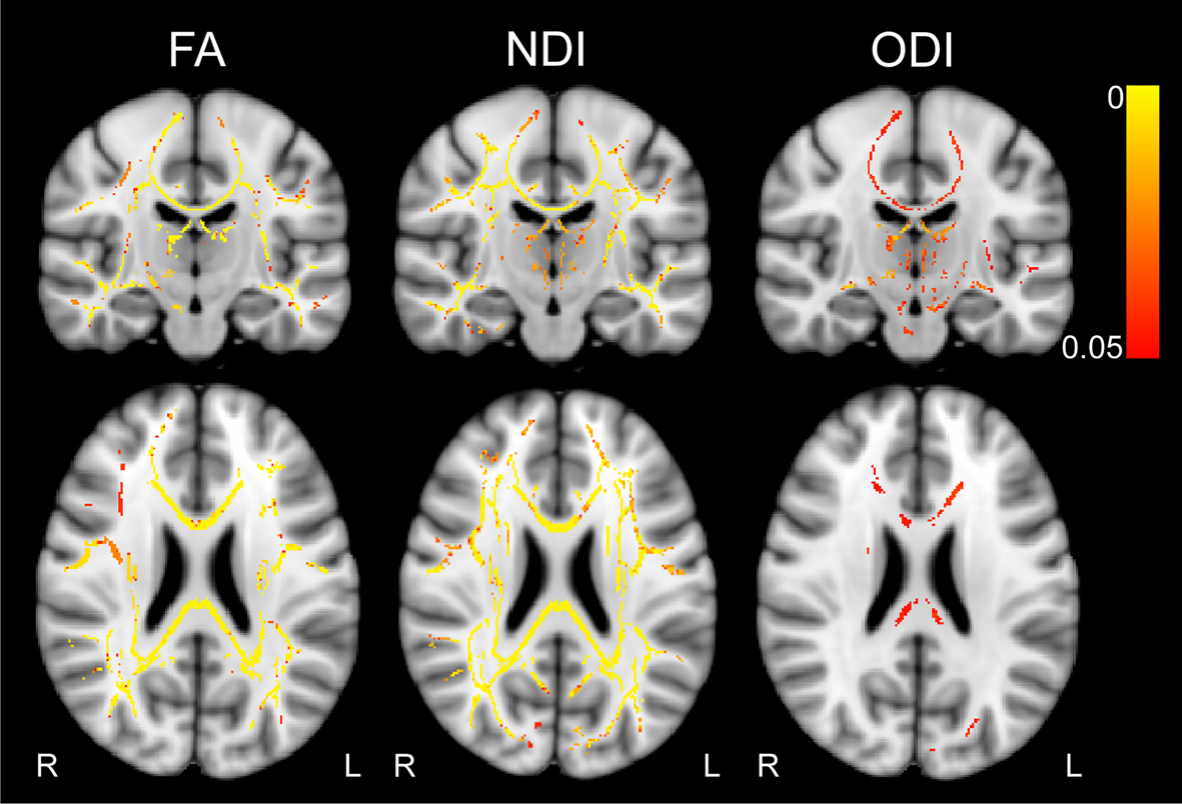
Results of tract-based spatial statistics analyses. Color bar indicates TFCE-corrected p-value, thresholded at p <.05. Contrast HC > MS for FA and NDI, and MS > HC for ODI. MNI coordinates: x = 90, y = 109, z = 95. FA, fractional anisotropy, NDI, neurite density index, ODI, orientation dispersion index, L, left, R, right, TFCE, threshold-free cluster enhancement.

First, we compared FA, NDI, ODI and IVF maps between HC and MS patients using TBSS. TFCE-based significance tests showed significantly higher FA (*p* <.05, Cohen’s d = 0.300) and NDI values (*p* <.05, Cohen’s d = 0.367) and lower ODI values (*p* <.05, Cohen’s d = 0.232) in HC compared to MS (**Figure 4**). No voxels showed significantly increased NDI or decreased ODI in MS compared to HC (*p* >.05). No significant group differences were observed regarding IVF (*p* >.05).

We next overlaid all WM masks of the SMATT on the mean FA skeleton, resulting in a mean motor skeleton. FA and NDI values of HC compared to MS were higher in a large amount of these motor WM tract voxels, i.e. 6.2% and 10.9%, respectively. Conversely, ODI was higher in MS patients compared to HC in 3% of the mean motor skeleton voxels.

To obtain more clarity regarding the regional effects, we computed the number and percentage of voxels showing group differences for each region of the SMATT atlas for FA, NDI and ODI maps (**Table 2**). Fibers exhibiting group differences in at least 10% of the region in either FA, NDI or ODI included right supplementary motor area (SMA), pre-SMA, ventral premotor cortex (PMv) and bilateral somatosensory cortex (S1) and M1.

**Table 2:** Number and percentage of voxels significantly differing between MS and HC in FA, NDI and ODI in SMATT regions.

SMATT label	FA (%)	NDI (%)	ODI (%)	Total
Right SMA	571 (11.28)	605 (11.95)	308 (6.09)	5061
Right M1	706 (8.17)	1348 (15.59)	377 (4.36)	8644
Right S1	363 (9.96)	391 (10.73)	198 (5.44)	3643
Left M1	481 (5.56)	1275 (14.75)	121 (1.40)	8644
Right PMv	379 (10.02)	350 (9.26)	84 (2.22)	3781
Right PMd	296 (7.00)	342 (8.09)	215 (5.08)	4230
Right pre-SMA	283 (4.95)	669 (11.70)	104 (1.82)	5720
Left SMA	240 (4.74)	410 (8.10)	263 (5.20)	5061
Left S1	182 (3.18)	681 (11.91)	47 (0.82)	5720
Left pre-SMA	143 (3.93)	287 (7.88)	140 (3.84)	3643
Left PMv	223 (5.90)	308 (8.15)	48 (1.27)	3781
Left PMd	10 (0.24)	80 (1.89)	101 (2.39)	4230
				
**TOTAL**	**3877 (6.24)**	**6746 (10.85)**	**2006 (3.09)**	**62158**

The last column contains the total number of voxels of the mean skeleton intersecting with each SMATT region. Contrast HC > MS for FA and NDI, and MS > HC for ODI, sorted decreasingly by the percentage of voxels showing significant group differences of each region, summed over FA, NDI and ODI.

MS, multiple sclerosis, HC, healthy control, FA, fractional anisotropy, NDI, neurite density index, ODI, orientation dispersion index, M1, primary motor cortex, SMA, supplementary motor area, S1, primary somatosensory cortex, PMd, dorsal premotor cortex, PMv, ventral premotor cortex.

**Figure 5:**
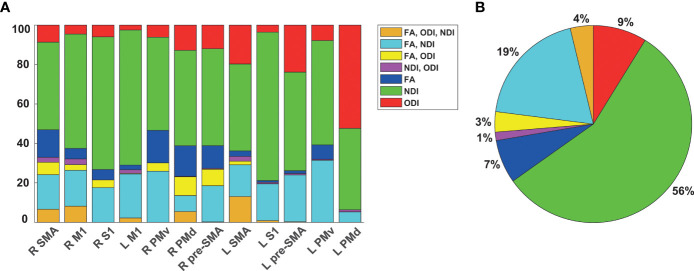
Comparison of diffusion parameters contributing to observed group differences. **(A)** Percentage of FA, NDI and ODI separately or combined showing group differences in the tract skeleton in intersection with SMATT regions. **(B)** Percentage averaged over regions with any significant group difference **(B)**. FA, fractional anisotropy, NDI, neurite density index, ODI, orientation dispersion index, SMA, supplementary motor area, M1, primary motor cortex, S1, primary somatosensory cortex, PMv, ventral premotor cortex. PMd, dorsal premotor cortex.

Next, we disentangled which combination of FA, NDI and ODI, or if one of the parameters alone contributed to observed group differences. **Figure 5A** depicts the percentage of all combinations and single parameters for each SMATT region, while the percentage averaged over all regions is presented in **Figure 5B**. Neurite density exclusively (56%) and in combination with FA (19%) accounted for the largest amount of differences, followed by ODI alone (9%). In **Supplementary Material S10**, we additionally provide results of a congruent analysis employing Johns Hopkins University (JHU) DTI-based WM atlas that also covers tracts outside the motor system (34–36) (**Supplementary Table S9** and **Supplementary Figure S5**).

## Discussion

In order to link microstructural integrity and cortical excitability, we applied a biophysical diffusion model on the basis of a multi-shell acquisition protocol. Lower microstructural integrity in the GM was linked to lower motor cortical excitability and lower cognitive-motor performance in MS. In the WM, we observed lower FA and NDI and higher ODI in MS patients compared to HC in all motor tracts. Voxel-wise group differences in FA strongly co-occurred with NDI differences. NDI and ODI appear sensitive for detection of neuropathological WM changes in MS patients undetected by FA.

### 1. Grey Matter Microstructural Integrity of the Motor System

#### 1a. Microstructural Correlates of Motor Excitability

Cortical excitability is frequently altered in MS patients following neuronal and axonal loss (4, 7). To unravel the link between microstructure and excitability, we applied NODDI, a biophysical model of microstructure. In contrast to classical DTI-based measures such as FA that are biased in areas of pronounced neurite orientation dispersion such as the GM, this tendency is mitigated in the NODDI model that is inherently more specific in depicting the cortical microstructure (17). We were interested in how motor cortical integrity quantified by NODDI parameters explains excitability levels as evaluated by the resting motor threshold. A hierarchical backward regression model revealed that lower NDI within M1 predicted higher motor thresholds, i.e. lower excitability in MS patients. This is well in line with the suggestion of axonal and myelin loss due to neuroinflammatory processes (2, 37). Furthermore, this shows the superiority of NDI in predicting excitability over local lesion load that was included in the model as a potential confounder. The HC model was not significant, however a trend of a positive relation between NDI and motor threshold was observable here as well. We suggest that higher variation of NDI led to a better model fit in the MS group as higher variance allows stronger correlations, while the model in general also fits to the HC group. In a supplementary analysis, we obtained NODDI and FA values within the hand area and observed comparable values to those of the whole M1 GM mask, again demonstrating a significant correlation between NDI and motor threshold in MS patients. When correlating FA and NODDI measures within the left M1 tract of the SMATT with motor threshold, we could not detect a significant relationship, well in line with previous studies (38, 39).

#### 1b. Microstructural Correlates of Cognitive-Motor Performance

We further assessed if microstructural integrity of the left M1 GM can also be linked to a behavioral outcome, i.e. cognitive-motor performance in the MS group. Hierarchical regression results indicated that patients with a lower neurite density showed decreased cognitive-motor performance as quantified by TMT-A, TMT-B and SDMT. For 9HPT, the regression model did not include significant predictors of interest. However, fewer patients completed this test and a trend for a positive correlation was detected between NDI and 9HPT scores. Previous results demonstrated a relation between lower cognitive performance and lower GM volume in regions relevant for demands of the utilized neuropsychological test (40), but also the whole brain (41). Lower magnetization transfer ratio was observed in motor regions for patients with lower 9HPT performance, likely reflecting pathological processes including inflammation, edema, demyelination and axonal loss (42, 43). The TMT measures multiple cognitive domains such as visuo-motor abilities and cognitive flexibility and it has been shown that precentral gyrus was activated in an fMRI-compatible TMT adaptation (44, 45). Hence, a lowered TMT performance linked to lower NDI in left M1 as seen here can be expected and underlines the suitability of the NODDI model for assessment of GM microstructural integrity.

### 2. White Matter Microstructural Integrity of the Motor System

In WM motor tracts, we detected decreased FA and NDI and increased ODI in patients with MS compared to HC, where the highest percentage of voxels showing group differences was in NDI. The strongest portion of sensorimotor tracts in intersection with group differences in the TBSS mean FA skeleton included WM tracts originating in right SMA and PMv. NDI was particularly lower in bilateral M1 tracts, whereas ODI was higher mainly in right SMA and S1 tracts of MS patients. GM atrophy in MS compared to HC in precentral and postcentral regions has been reported previously (46). The recently observed M1 and SMA atrophy in patients with disability progression after 10 years make it plausible that connecting WM tracts show abnormalities due to primary or secondary causes as well (47). In further support of this view, lower FA was observed in the corticospinal tract of MS patients, and higher mean, axial and radial diffusivity was linked to lower M1 thickness (48). A small sample-sized first NODDI study reported decreased NDI and increased ODI in the normal-appearing WM of MS patients (49). Higher ODI indicates fiber coherence loss and can possibly be explained by an increase in compensative axonal sprouting or branching (50). Regarding NDI, a decrease in MS is expected due to the demyelinating, inflammatory and neuro-axonal pathology of the disease (2).

Importantly, low FA values could be caused by lower NDI, higher ODI, or a combination of both (17), such that it is of great relevance to disentangle if group effects appear for these parameters exclusively in a voxel, or if they potentially overlap, which has not yet been systematically quantified in MS research to our knowledge. This allows a more concrete inference on the type of microstructure change in contrast to assumptions solely based on FA. On average, 56% of voxels showing significant group differences in intersection with the mean FA skeleton were different exclusively in NDI, whereas 19% were different simultaneously in FA and NDI, 9% in ODI only and 7% in FA only. Our findings suggest that FA is capable of detecting parts of the pathological alterations that can mainly be retraced to NDI decreases in MS. The NODDI model therefore allows to capture further microstructure changes in MS in addition to its inherently higher specificity to the underlying pathology (17). These TBSS results support previous findings based on voxel-wise comparisons (49) and further disentangle that the lower FA signal is predominantly caused by reduced neurite density in sensorimotor WM tracts of MS patients.

## Limitations and Prospect

We provide novel insights regarding the applicability of the NODDI model to assess microstructure in both GM and WM. Our suggestion that neurite density is also linked to motor cortical excitability in healthy participants needs to be further examined in future studies including larger sample sizes or a subject group with a higher variability in age, as individuals of higher age typically show higher resting motor thresholds and increasing neuronal loss (51, 52). It is of note that the established microstructural integrity-excitability link in our study sample does not appear to be disease-specific, as the regression slopes were not significantly different between healthy individuals and patients. We rather assume that this relationship is existent independent from disease activity, but that multiple sclerosis amplifies the correlation due to the increased variance of NDI. The present work did not examine a link between microstructure and subscales of the EDSS, but instead focused on neuropsychological test scores. Future studies tackling this relation would be of strong interest. Furthermore, details on spinal cord involvement were not incorporated, which would be an interesting research question to revisit in future works. Not all MS patients completed the 9HPT, which likely lowered statistical power. The link between neuropsychological test performance and NODDI parameters could in future be expanded to other cognitive domains and brain regions analyzing larger patient samples. It would be particularly interesting to observe potential effects of motor and cognitive training or medication on beneficial adaptations in microstructural integrity (53). In this study, however, neuropsychological performance was not assessed in the healthy sample, such that the relation to microstructural integrity needs to be established for healthy participants in future work. How microstructural state as evaluated by biophysical diffusion models is connected to functional adaptive and maladaptive mechanisms of brain networks would be an interesting question to revisit using functional MRI (54, 55). A translational approach linking NODDI in different animal models of MS to human patients remains to be further pursued as was done with a cuprizone model of demyelination in mice and by applying DTI (15, 19, 56). Furthermore, differentiating the evolution of microstructure in MS compared to healthily aging individuals is of interest in longitudinal investigations (57).

## Conclusion

In this work, we demonstrate that lower neurite density in left M1 is linked to decreased motor cortical excitability and impaired cognitive-motor performance in patients with MS. We showed that lower neurite density and higher orientation dispersion are characteristic in the WM of MS patients compared to HC, and that these markers are more sensitive to pathological alterations than the classical DTI measure FA. These results suggest that advanced biophysical diffusion models are of great relevance for prediction of neurodegenerative processes and disease progression. Our findings establish a link between microstructure imaging of the grey matter and excitability in the motor system.

## Data Availability Statement

The raw data supporting the conclusions of this article will be made available by the authors, without undue reservation.

## Ethics Statement

The studies involving human participants were reviewed and approved by Ethics Committee of the State Medical Association of Rhineland-Palatinate. The patients/participants provided their written informed consent to participate in this study.

## Author Contributions

Conceptualized the study: AR, MM, SG, SB, and SM. Acquired and analyzed participants’ data: AR, KM, MM, and SG. Original drafting of the manuscript: AR. Interpretation of data, review and editing of the manuscript: AR, KM, DC, GG-E,VF, EE, JK, SB, SM, MM, and SG. All authors contributed to the article and approved the submitted version.

## Conflict of Interest

JK received honoraria for lecturing from Biogen, Novartis, Merck Serono, Sanofi-Genzyme, Roche, Mylan and Teva, and financial research support from Sanofi Genzyme. SB has received honoraria and compensation for travel from Biogen Idec, Merck Serono, Novartis, Sanofi-Genzyme and Roche. SM has received honoraria for lecturing and travel expenses for attending meetings from Almirall, Amicus Therapeutics Germany, Bayer Health Care, Biogen, Celgene, Diamed, Genzyme, MedDay Pharmaceuticals, Merck Serono, Novartis, Novo Nordisk, ONO Pharma, Roche, Sanofi-Aventis, Chugai Pharma, QuintilesIMS, and Teva. His research is funded by the German Ministry for Education and Research (BMBF), Deutsche Forschungsgemeinschaft (DFG), Else Kröner Fresenius Foundation, German Academic Exchange Service, Hertie Foundation, Interdisciplinary Center for Clinical Studies (IZKF) Muenster, German Foundation Neurology, and by Almirall, Amicus Therapeutics Germany, Biogen, Diamed, Fresenius Medical Care, Genzyme, Merck Serono, Novartis, ONO Pharma, Roche, and Teva.

The remaining authors declare that the research was conducted in the absence of any commercial or financial relationships that could be construed as a potential conflict of interest.

## Publisher’s Note

All claims expressed in this article are solely those of the authors and do not necessarily represent those of their affiliated organizations, or those of the publisher, the editors and the reviewers. Any product that may be evaluated in this article, or claim that may be made by its manufacturer, is not guaranteed or endorsed by the publisher.

## Abbreviations

AD, axial diffusivity; APB, abductor pollicis brevis; AMICO, Accelerated Microstructure Imaging *via* Convex Optimization; DTI, diffusion tensor imaging; DWI, diffusion-weighted imaging; PMd, dorsal premotor cortex; TE, echo time; EMG, electromyography; EDSS, Expanded Disability Status Scale; FOV, field of view; FDI, first dorsal interosseous; FLAIR, fluid attenuated inversion recovery; FA, fractional anisotropy; GM, grey matter; HC, healthy controls; HMAT, Human Motor Area Template; TI, inversion time; IVF, isotropic volume fraction; JHU, Johns Hopkins University; L, left; LST, lesion segmentation toolbox; MD, mean diffusivity; MEP, motor evoked potential; MS, multiple sclerosis; NDI, neurite density index; NODDI, neurite orientation dispersion and density imaging; 9HPT, Nine Hole Peg Test; ODI, orientation dispersion index; M1, primary motor cortex; S1, primary somatosensory cortex; RD, radial diffusivity; TR, repetition time; RRMS, relapsing-remitting MS; R, right; SMATT, sensorimotor area tract template; SD, standard deviation; SMA, supplementary motor area; SDMT, Symbol Digit Modalities Task; TFCE, threshold-free cluster enhancement; TBSS, tract-based spatial statistics; TMT-A, Trail Making Test part A; TMT-B, Trail Making Test part B; TMS, transcranial magnetic stimulation; PMv, ventral premotor cortex; WM, white matter.
