# Supplementary material for: Linking Microstructural Integrity and Motor Cortex Excitability in Multiple Sclerosis
Source: Front Immunol. 2021 Oct 12;12:748357. doi: 10.3389/fimmu.2021.748357 (PMC8546169; doi:10.3389/fimmu.2021.748357)
Supplement: Supplementary file 1 [file DataSheet_1.doc]

***Supplementary Material***

*S1. Further medication and comorbidities of MS patients*

Supplementary Table 1. Further medication and comorbidities of MS patient sample.

| Further drugs | |  |
| --- | --- | --- |
|  | Escitalopram | 3 |
|  | Gabapentin | 2 |
|  | Pregabalin | 2 |
|  | Duluxetine | 1 |
|  | Quetiapine | 1 |
|  | Sertraline | 1 |
|  | Valproate | 1 |
|  | Venlafaxine | 1 |
| Comorbidities | |  |
|  | Hypertension | 9 |
|  | Hypothyreosis | 4 |
|  | Depression | 4 |
|  | Asthma | 3 |
|  | Anxiety disorder | 2 |
|  | Bipolar disorder | 2 |
|  | Breast cancer | 1 |
|  | Factor V Leiden | 1 |
|  | Fibromyalgia | 1 |
|  | Hyperlipidemia | 1 |
|  | Lung embolism | 1 |
|  | Neurodermitis | 1 |
|  | Raynaud’s disease | 1 |
|  | Ulcerative colitis | 1 |

*Note:* A total of n = 9 patients was administered one or more of the listed drugs and a total of n = 27 patients presented with one or more comorbidity.

*S2. Group differences in motor threshold*

We investigated group differences of motor threshold between MS and HC. Excitability levels, i.e. motor thresholds were non-significantly higher in MS patients compared to HC (t(97) = 0.78, *p* > .05; MS group: mean [SD] = 58.06 [10.60]; HC group: mean [SD] = 56.55 [8.63]). Levene’s test did not reveal a significant difference in the spread of motor thresholds (F(1,97) = 1.576; *p* = .212).

Higher motor thresholds, i.e. lower excitability, in MS than HC have been reported previously, particularly for the secondary progressive disease type and in patients in the relapsing phase ^1,2^. Decreased motor cortical excitability in MS patients potentially is a consequence of neuronal loss, axonal scarcity or synaptic down-scaling of the cortico-cortical inputs ^3–6^. The motor thresholds did not statistically differ between the addressed groups in our study, however showed a trending increase in MS.

*S3. Group differences in FA, NDI, ODI and IVF in left M1 GM*

Group comparisons in FA, NDI, ODI and IVF in the GM of left M1 all did not reveal any significant group differences (all *p* > .05, Table S2). Variability of NDI in MS patients was significantly higher than in HC (F(1,97) = 4.25, *p* = .04, Supplementary Table 6).

All diffusion measures did not significantly depict group differences, but in line with a previous study, trending lower ODI in MS compared to HC was observable ^7^. A decrease in the GM ODI could indicate degeneration of single neurites, resulting in dispersion reductions ^8,9^. Presumably, both a larger sample size and a longer disease duration would amplify these trending differences in motor threshold and NODDI parameters between MS and HC.

Supplementary Table 2. FA, NDI, ODI and IVF in left M1 GM in MS compared to HC

| **Diffusion parameter** | **MS (mean [SD])** | **HC (mean [SD])** | **t-test** | **Levene’s test** |
| --- | --- | --- | --- | --- |
| **FA** | m = 0.1539 [0.0204] | m = 0.1515 [0.098] | t(97) = -0.61; *p* > .05 | F(1,97) = 0.14, *p* > .05 |
| **NDI** | m = 0.3915 [0.0393] | m = 0.3916 [0.0321] | t(97) = 0.01; *p* > .05 | F(1,97) = 4.25, *p* = .04 |
| **ODI** | m = 0.5119 [0.0344] | m = 0.5195 [0.0330] | t(97) = 1.11; *p* > .05 | F(1,97) = 2.3*e^-4^, *p* > .05 |
| **IVF** | m = 0.2889 [0.1112] | m = 0.2820 [0.0825] | t(97) = -0.35; *p* > .05 | F(1,97) = 2.78, *p* > .05 |

*Note*: T-test for comparison of group differences in each diffusion parameter, Levene’s test for testing of variance inequality. Abbreviations: FA, fractional anisotropy, NDI, neurite density index, ODI, orientation dispersion index, IVF, isotropic volume fraction, M1, primary motor cortex, GM, grey matter, MS, multiple sclerosis, HC, healthy control; SD, standard deviation.

*S4. Correlation coefficients of the correlation between left M1 diffusion measures and motor threshold*

Within the GM of both MS and HC, diffusion measures were significantly interrelated (Supplementary Figure 1). FA and NDI were positively interrelated, but negatively correlated to ODI and IVF. In MS patients, higher NDI was significantly linked to lower motor threshold; HC exhibited a similar trend.


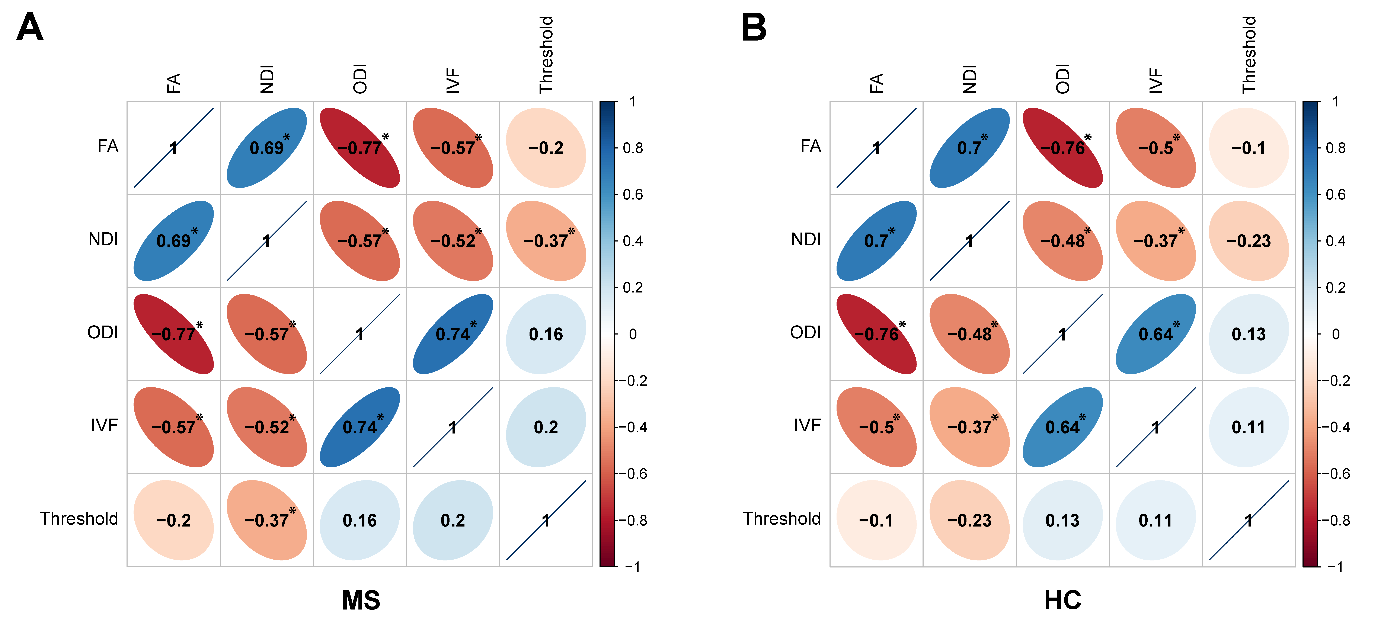


Supplementary Figure 1. Correlation coefficients of the correlation between left M1 GM FA, NDI, ODI, IVF and motor threshold in (A) MS and (B) HC. * p < .05 (FDR corrected). Abbreviations: M1, primary motor cortex, GM, grey matter, FA, fractional anisotropy, NDI, neurite density index, ODI, orientation dispersion index, IVF, isotropic volume fraction, MS, multiple sclerosis, HC, healthy controls.

*S5.* *Detailed results of hierarchical regression analysis of motor threshold in MS*

Supplementary Table 3. Results of hierarchical regression analysis of motor threshold in MS

|  | **Regression coefficient** | **Standard error** | **Beta coefficient** | **t statistic** | ***p* value** |
| --- | --- | --- | --- | --- | --- |
| **Model 1** |  |  |  |  |  |
| Age | 0.006 | 0.152 | 0.007 | 0.042 | .967 |
| Gender | 4.577 | 3.431 | 0.212 | 1.334 | .189 |
| M1 lesion load | 4.110 | 3.042 | 0.209 | 1.351 | .184 |
| ODI | -59.439 | 83.964 | -0.193 | -0.708 | .483 |
| IVF | 15.148 | 23.349 | 0.159 | 0.649 | .520 |
| FA | -35.262 | 134.085 | -0.068 | -0.263 | .794 |
| NDI | -94.881 | 54.737 | -0.352 | -1.733 | .090 |
|  | F(7,42) = 1.563; *p* = .173; adjusted r² = .074 | | | | |
| **Model 2** |  |  |  |  |  |
| Gender | 4.608 | 3.308 | 0.213 | 1.393 | .171 |
| M1 lesion load | 4.135 | 2.948 | 0.210 | 1.403 | .168 |
| ODI | -59.768 | 82.612 | -0.195 | -0.723 | .473 |
| IVF | 15.564 | 20.860 | 0.163 | 0.746 | .460 |
| FA | -35.547 | 132.346 | -0.068 | -0.269 | .790 |
| NDI | -94.511 | 53.377 | -0.350 | -1.771 | .084 |
|  | F(6,43) = 1.866; *p* = .109; adjusted r² = .096 | | | | |
| **Model 3** |  |  |  |  |  |
| Gender | 3.647 | 2.958 | 0.185 | 1.233 | .224 |
| ODI | -48.589 | 83.095 | -0.158 | -0.585 | .562 |
| IVF | 6.536 | 20.039 | 0.069 | 0.326 | .746 |
| FA | -15.899 | 132.991 | -0.031 | -0.120 | .905 |
| NDI | -95.449 | 53.940 | -0.354 | -1.770 | .084 |
|  | F(5,44) = 1.812; *p* = .130; adjusted r² = .077 | | | | |
| **Model 4** |  |  |  |  |  |
| ODI | -25.685 | 81.459 | -0.084 | -0.315 | .754 |
| IVF | 7.612 | 20.135 | 0.080 | 0.378 | .707 |
| FA | 31.527 | 128.041 | 0.061 | 0.246 | .807 |
| NDI | -111.908 | 52.564 | -0.415 | -2.129 | .039 |
|  | F(4,45) = 1.864; *p* = .133; adjusted r² = .066 | | | | |
| **Model 5** |  |  |  |  |  |
| IVF | 3.939 | 16.262 | 0.041 | 0.242 | .810 |
| FA | 54.543 | 104.161 | 0.105 | 0.524 | .603 |
| NDI | -112.695 | 51.988 | -0.418 | -2.168 | .035 |
|  | F(3,46) =2.501; *p* = .071; adjusted r² = .084 | | | | |
| **Model 6** |  |  |  |  |  |
| FA | 45.940 | 96.933 | 0.088 | 0.474 | .638 |
| NDI | -115.445 | 50.222 | -0.428 | -2.299 | .026 |
|  | F(2,47) = 3.798; *p* = .030; adjusted r² = .103 | | | | |
| **Model 7** |  |  |  |  |  |
| NDI | -99.095 | 36.201 | -0.367 | -2.737 | .009 |
|  | F(1,48) = 7.493; *p* = .009; adjusted r² = .117 | | | | |

*Note*: Abbreviations: MS, multiple sclerosis, M1, primary motor cortex, ODI, orientation dispersion index, IVF, isotropic volume fraction, FA, fractional anisotropy, NDI, neurite density index.

*S6.* *Detailed results of hierarchical regression analysis of motor threshold in HC*

Supplementary Table 4. Results of hierarchical regression analysis of motor threshold in HC

|  | **Regression coefficient** | **Standard error** | **Beta coefficient** | **t statistic** | ***p* value** |
| --- | --- | --- | --- | --- | --- |
| **Model 1** |  |  |  |  |  |
| Age | 0.023 | 0.157 | 0.025 | 0.149 | .882 |
| Gender | 1.351 | 2.906 | 0.079 | 0.465 | .644 |
| FA | 100.837 | 128.311 | 0.231 | 0.786 | .436 |
| IVF | 1.212 | 22.554 | 0.012 | 0.054 | .957 |
| ODI | 41.867 | 69.387 | 0.160 | 0.603 | .549 |
| NDI | -91.277 | 56.875 | -.339 | -1.605 | .116 |
|  | F(6,42) = 0.592; *p* = .735; adjusted r² = -.054 | | | | |
| **Model 2** |  |  |  |  |  |
| Gender | 1.277 | 2.830 | 0.075 | 0.451 | .654 |
| FA | 101.521 | 126.762 | 0.233 | 0.801 | .428 |
| IVFI | 2.584 | 20.350 | .025 | 0.127 | .900 |
| ODI | 40.253 | 67.751 | 0.154 | 0.594 | .556 |
| NDI | -90.654 | 56.073 | -0.337 | -1.617 | .113 |
|  | F(5,43) = 0.723; *p* = .610; adjusted r² = -.030 | | | | |
| **Model 3** |  |  |  |  |  |
| FA | 114.957 | 122.095 | 0.263 | 0.942 | .352 |
| IVFI | 0.846 | 19.800 | 0.008 | 0.043 | .966 |
| ODI | 42.934 | 66.876 | 0.164 | 0.642 | .524 |
| NDI | -90.221 | 55.555 | -0.335 | -1.624 | .112 |
|  | F(4,44) = 0.868; *p* = .491; adjusted r² = -.011 | | | | |
| **Model 4** |  |  |  |  |  |
| IVF | 1.526 | 19.762 | 0.015 | 0.077 | .939 |
| ODI | 3.669 | 52.215 | 0.014 | 0.070 | .944 |
| NDI | -59.257 | 44.718 | -0.220 | -1.325 | .192 |
|  | F(3,45) = 0.864; *p* = .467; adjusted r² = -.009 | | | | |
| **Model 5** |  |  |  |  |  |
| ODI | 5.935 | 42.718 | 0.023 | 0.139 | .890 |
| NDI | -59.598 | 44.016 | -.221 | -1.354 | .182 |
|  | F(2,46) = 1.322; *p* = .277; adjusted r² = .013 | | | | |
| **Model 6** |  |  |  |  |  |
| NDI | -62.537 | 38.197 | -0.232 | -1.637 | .108 |
|  | F(1,47) = 2.681; *p* = .108; adjusted r² = .034 | | | | |

*Note*: Abbreviations: HC, healthy controls, FA, fractional anisotropy, IVF, isotropic volume fraction, ODI, orientation dispersion index, NDI, neurite density index.

*S7. Detailed results of neuropsychological analyses*


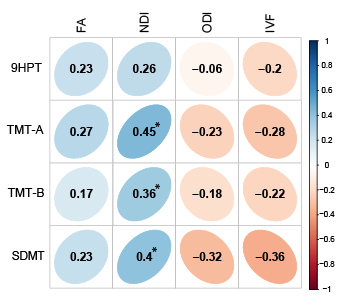


Supplementary Figure 2. Correlation coefficients of the correlation between left M1 GM FA, NDI, ODI, IVF and each 9HPT, TMT-A, TMT-B and SDMT in MS patients. * p < .05 (FDR corrected). Abbreviations: M1, primary motor cortex, GM, grey matter, FA, fractional anisotropy, NDI, neurite density index, ODI, orientation dispersion index, IVF, isotropic volume fraction, 9HPT, Nine Hole Peg Test, TMT-A, Trail Making Test part A, TMT-B, Trail Making Test part B, SDMT, Symbol Digit Modalities Test, MS, multiple sclerosis.

Supplementary Table 5. Results of hierarchical regression analysis of 9HPT

|  | **Regression coefficient** | **Standard error** | **Beta coefficient** | **t statistic** | ***p* value** |
| --- | --- | --- | --- | --- | --- |
| **Model 1** |  |  |  |  |  |
| Age | -0.052 | 0.025 | -0.424 | -2.074 | .049 |
| Gender | 0.198 | 0.643 | 0.069 | 0.307 | .761 |
| M1 lesion load | -0.567 | 0.482 | -0.265 | -1.178 | .251 |
| ODI | 24.673 | 17.248 | 0.595 | 1.430 | .166 |
| IVF | 0.880 | 5.522 | 0.062 | 0.159 | .875 |
| FA | 37.388 | 27.311 | 0.516 | 1.369 | .184 |
| NDI | 1.343 | 10.316 | 0.033 | 0.130 | .898 |
|  | F(7,23) = 1.473; *p* = .226; adjusted r² = .099 | | | | |
| **Model 2** |  |  |  |  |  |
| Age | -0.051 | 0.024 | -0.414 | -2.091 | .047 |
| M1 lesion load | -0.559 | 0.472 | -0.261 | -1.185 | .248 |
| ODI | 23.522 | 16.516 | 0.567 | 1.424 | .167 |
| IVF | 0.505 | 5.284 | 0.036 | 0.096 | .925 |
| FA | 35.866 | 26.347 | 0.495 | 1.361 | .186 |
| NDI | 2.297 | 9.650 | 0.056 | 0.238 | .814 |
|  | F(6,24) = 1.769; *p* = .148; adjusted r² = .133 | | | | |
| **Model 3** |  |  |  |  |  |
| Age | -0.053 | 0.024 | -0.436 | -2.196 | .038 |
| ODI | 17.785 | 15.918 | 0.429 | 1.117 | .274 |
| IVF | -0.666 | 5.232 | -0.047 | -0.127 | .900 |
| FA | 20.717 | 23.223 | 0.286 | 0.892 | .381 |
| NDI | 7.536 | 8.648 | 0.184 | 0.871 | .392 |
|  | F(5,25) = 1.813; *p* = .147; adjusted r² = .119 | | | | |
| **Model 4** |  |  |  |  |  |
| Age | -0.058 | 0.024 | -0.476 | -2.426 | .022 |
| IVF | 3.217 | 3.930 | 0.227 | 0.818 | .421 |
| FA | 8.332 | 20.505 | 0.115 | 0.406 | .688 |
| NDI | 9.053 | 8.581 | 0.221 | 1.055 | .301 |
|  | F(4,26) = 1.935; *p* = .134; adjusted r² = .111 | | | | |
| **Model 5** |  |  |  |  |  |
| Age | -0.050 | 0.022 | -0.409 | -2.310 | .029 |
| FA | -2.550 | 15.514 | -0.035 | -0.164 | .871 |
| NDI | 9.124 | 8.528 | 0.223 | 1.070 | .294 |
|  | F(3,27) = 2.386; *p* = .091; adjusted r² = .122 | | | | |
| **Model 6** |  |  |  |  |  |
| Age | -0.049 | 0.021 | -0.401 | -2.385 | .024 |
| NDI | 8.324 | 6.879 | 0.204 | 1.210 | .236 |
|  | F(2,28) = 3.694; *p* = .038; adjusted r² = .152 | | | | |
| **Model 7** |  |  |  |  |  |
| Age | -0.050 | 0.021 | -0.409 | -2.415 | .022 |
|  | F(1,29) = 5.831; *p* = .022; adjusted r² = .139 | | | | |

*Note*: Abbreviations: 9HPT, Nine Hole Peg Test, M1, primary motor cortex, ODI, orientation dispersion index, IVF, isotropic volume fraction, FA, fractional anisotropy, NDI, neurite density index.

Supplementary Table 6. Results of hierarchical regression analysis of TMT-A

|  | **Regression coefficient** | **Standard error** | **Beta coefficient** | **t statistic** | ***p* value** |
| --- | --- | --- | --- | --- | --- |
| **Model 1** |  |  |  |  |  |
| Age | -0.005 | 0.019 | -0.046 | -0.272 | .787 |
| Gender | 0.179 | 0.478 | 0.072 | 0.374 | .711 |
| M1 lesion load | -0.469 | 0.369 | -0.213 | -1.272 | .212 |
| ODI | 12.777 | 10.947 | 0.328 | 1.167 | .251 |
| FA | 1.106 | 16.458 | 0.018 | 0.067 | .947 |
| IVF | -3.705 | 3.227 | -0.292 | -1.148 | .258 |
| NDI | 12.037 | 7.484 | 0.332 | 1.608 | .117 |
|  | F(7,36) = 1.923; *p* = .094; adjusted r² = .131 | | | | |
| **Model 2** |  |  |  |  |  |
| Gender | 0.150 | 0.460 | 0.060 | 0.325 | .747 |
| M1 lesion load | -0.482 | 0.360 | -0.219 | -1.338 | .189 |
| ODI | 13.119 | 10.738 | 0.337 | 1.222 | .230 |
| FA | 1.485 | 16.192 | 0.024 | 0.092 | .927 |
| IVF | -4.095 | 2.855 | -0.323 | -1.434 | .160 |
| NDI | 11.727 | 7.304 | 0.324 | 1.606 | .117 |
|  | F(6,37) =2.289; *p* = .056; adjusted r² = .152 | | | | |
| **Model 3** |  |  |  |  |  |
| M1 lesion load | -0.476 | 0.356 | -0.216 | -1.337 | .189 |
| ODI | 12.656 | 10.517 | 0.325 | 1.203 | .236 |
| FA | 0.996 | 15.931 | 0.016 | 0.062 | .950 |
| IVF | -4.427 | 2.635 | -0.349 | -1.680 | .101 |
| NDI | 12.373 | 6.945 | 0.342 | 1.782 | .083 |
|  | F(5,38) = 2.791; *p* = .030; adjusted r² = .172 | | | | |
| **Model 4** |  |  |  |  |  |
| ODI | 9.095 | 10.276 | 0.233 | 0.885 | .382 |
| FA | -5.886 | 15.229 | -0.094 | -0.387 | .701 |
| IVF | -4.883 | 2.639 | -0.385 | -1.851 | .072 |
| NDI | 15.574 | 6.585 | 0.430 | 2.365 | .023 |
|  | F(4,39) = 2.982; *p* = .031; adjusted r² = .156 | | | | |
| **Model 5** |  |  |  |  |  |
| FA | -13.576 | 12.473 | -0.217 | -1.088 | .283 |
| IVF | -3.464 | 2.090 | -0.273 | -1.657 | .105 |
| NDI | 16.101 | 6.540 | 0.445 | 2.462 | .018 |
|  | F(3,40) = 3.735; *p* = .019; adjusted r² = .160 | | | | |
| **Model 6** |  |  |  |  |  |
| IVF | -2.491 | 1.893 | -0.197 | -1.316 | .196 |
| NDI | 12.085 | 5.412 | 0.334 | 2.233 | .031 |
|  | F(2,41) = 4.987; *p* = .012; adjusted r² = .156 | | | | |
| **Model 7** |  |  |  |  |  |
| NDI | 14.565 | 5.117 | 0.402 | 2.846 | .007 |
|  | F(1,42) = 8.102; *p* = .007; adjusted r² = .142 | | | | |

*Note*: Abbreviations: TMT-A, Trail Making Test part A, M1, primary motor cortex, ODI, orientation dispersion index, FA, fractional anisotropy, IVF, isotropic volume fraction, NDI, neurite density index.

Supplementary Table 7. Results of hierarchical regression analysis of TMT-B

|  | **Regression coefficient** | **Standard error** | **Beta coefficient** | **t statistic** | ***p* value** |
| --- | --- | --- | --- | --- | --- |
| **Model 1** |  |  |  |  |  |
| Age | -0.018 | 0.018 | -0.173 | -0.999 | .324 |
| Gender | 0.253 | 0.446 | 0.112 | 0.566 | .575 |
| M1 lesion load | -0.325 | 0.344 | -0.163 | -0.944 | .351 |
| ODI | 4.940 | 10.224 | 0.140 | 0.483 | .632 |
| FA | -1.758 | 15.370 | -0.031 | -0.114 | .910 |
| IVF | -0.388 | 3.014 | -0.034 | -0.129 | .898 |
| NDI | 11.692 | 6.990 | 0.357 | 1.673 | .103 |
|  | F(7,36) = 1.485; *p* = .204; adjusted r² = .073 | | | | |
| **Model 2** |  |  |  |  |  |
| Age | -0.016 | 0.017 | -0.151 | -0.904 | .372 |
| M1 lesion load | -0.320 | 0.341 | -0.161 | -0.939 | .354 |
| ODI | 4.345 | 10.075 | 0.123 | 0.431 | .669 |
| FA | -2.379 | 15.189 | -0.042 | -0.157 | .876 |
| IVF | -1.090 | 2.722 | -0.095 | -0.400 | .691 |
| NDI | 12.595 | 6.742 | 0.384 | 1.868 | .070 |
|  | F(6,37) = 1.711; *p* = .146; adjusted r² = .090 | | | | |
| **Model 3** |  |  |  |  |  |
| M1 lesion load | -0.365 | 0.336 | -0.183 | -1.085 | .285 |
| ODI | 5.639 | 9.949 | 0.160 | 0.567 | .574 |
| FA | -0.960 | 15.072 | -0.017 | -0.064 | .950 |
| IVF | -2.065 | 2.492 | -0.180 | -0.829 | .412 |
| NDI | 11.289 | 6.570 | 0.345 | 1.718 | .094 |
|  | F(5,38) = 1.899; *p* = .117; adjusted r² = .095 | | | | |
| **Model 4** |  |  |  |  |  |
| ODI | 2.907 | 9.647 | 0.083 | 0.301 | .765 |
| FA | -6.241 | 14.296 | -0.110 | -0.437 | .665 |
| IVF | -2.415 | 2.477 | -0.211 | -0.975 | .335 |
| NDI | 13.745 | 6.182 | 0.420 | 2.223 | .032 |
|  | F(4,39) = 2.070; *p* = .103; adjusted r² = .091 | | | | |
| **Model 5** |  |  |  |  |  |
| FA | -8.699 | 11.607 | -0.154 | -0.749 | .458 |
| IVF | -1.962 | 1.945 | -0.171 | -1.009 | .319 |
| NDI | 13.913 | 6.086 | 0.425 | 2.286 | .028 |
|  | F(3,40) = 2.794; *p* = .053; adjusted r² = .111 | | | | |
| **Model 6** |  |  |  |  |  |
| IVF | -1.339 | 1.749 | -0.117 | -0.766 | .448 |
| NDI | 11.340 | 4.998 | 0.346 | 2.269 | .029 |
|  | F(2,41) = 3.952; *p* = .027; adjusted r² = .121 | | | | |
| **Model 7** |  |  |  |  |  |
| NDI | 12.672 | 4.662 | 0.387 | 2.718 | .009 |
|  | F(1,42) = 7.390; *p* = .009; adjusted r² = .129 | | | | |

*Note*: Abbreviations: TMT-B, Trail Making Test part B, M1, primary motor cortex, ODI, orientation dispersion index, FA, fractional anisotropy, IVF, isotropic volume fraction, NDI, neurite density index.

Supplementary Table 8. Results of hierarchical regression analysis of SDMT

|  | **Regression coefficient** | **Standard error** | **Beta coefficient** | **t statistic** | ***p* value** |
| --- | --- | --- | --- | --- | --- |
| **Model 1** |  |  |  |  |  |
| Age | 0.019 | 0.016 | 0.213 | 1.196 | .239 |
| Gender | 0.096 | 0.369 | 0.046 | 0.263 | .794 |
| M1 lesion load | -0.001 | 0.003 | -0.056 | -0.359 | .722 |
| FA | -13.072 | 13.618 | -0.253 | -0.960 | .343 |
| ODI | -2.744 | 9.135 | -0.087 | -0.300 | .766 |
| IVF | -3.422 | 2.643 | -0.346 | -1.295 | .203 |
| NDI | 10.051 | 5.565 | 0.365 | 1.806 | .079 |
|  | F(7,36) = 1.915; *p* = .095; adjusted r² = .127 | | | | |
| **Model 2** |  |  |  |  |  |
| Age | 0.021 | 0.015 | 0.228 | 1.374 | .177 |
| M1 lesion load | -0.001 | 0.003 | -0.061 | -0.400 | .691 |
| FA | -12.909 | 13.436 | -0.250 | -0.961 | .343 |
| ODI | -2.860 | 9.012 | -0.090 | -0.317 | .753 |
| IVF | -3.663 | 2.447 | -0.370 | -1.497 | .143 |
| NDI | 10.068 | 5.496 | 0.365 | 1.832 | .075 |
|  | F(6,37) = 2.279; *p* = .056; adjusted r² = .148 | | | | |
| **Model 3** |  |  |  |  |  |
| Age | 0.020 | 0.015 | 0.221 | 1.355 | .183 |
| FA | -14.542 | 12.663 | -0.282 | -1.148 | .258 |
| ODI | -3.671 | 8.686 | -0.116 | -0.423 | .675 |
| IVF | -3.637 | 2.420 | -0.368 | -1.503 | .141 |
| NDI | 10.754 | 5.165 | 0.390 | 2.082 | .044 |
|  | F(5,38) = 2.762; *p* = .031; adjusted r² = .167 | | | | |
| **Model 4** |  |  |  |  |  |
| FA | -14.867 | 12.792 | -0.288 | -1.162 | .252 |
| ODI | -4.900 | 8.728 | -0.155 | -0.561 | .578 |
| IVF | -2.147 | 2.178 | -0.217 | -0.986 | .330 |
| NDI | 11.331 | 5.201 | 0.411 | 2.179 | .035 |
|  | F(4,39) = 2.932; *p* = .032; adjusted r² = .149 | | | | |
| **Model 5** |  |  |  |  |  |
| ODI | -0.951 | 7.161 | 0.030 | 0.133 | .455 |
| IVF | -2.418 | 2.174 | -0.244 | -1.112 | .273 |
| NDI | 8.316 | 4.527 | 0.302 | 1.837 | .073 |
|  | F(3,40) = 3.430; *p* = .026; adjusted r² = .142 | | | | |
| **Model 6** |  |  |  |  |  |
| IVF | -2.217 | 1.547 | -0.224 | -1.434 | .159 |
| NDI | 8.155 | 4.309 | 0.296 | 1.892 | .065 |
|  | F(2,41) = 5.259; *p* = .009; adjusted r² = .162 | | | | |
| **Model 7** |  |  |  |  |  |
| NDI | 11.061 | 3.849 | 0.401 | 2.874 | .006 |
|  | F(1,42) = 8.260; *p* = .006; adjusted r² = .142 | | | | |

*Note*: Abbreviations: SDMT, Symbol Digit Modalities Test, M1, primary motor cortex, ODI, orientation dispersion index, FA, fractional anisotropy, IVF, isotropic volume fraction, NDI, neurite density index.

*S8. Correlation analysis of FA and NODDI measures within the hand area and motor threshold*

As a supplementary analysis, we applied a 12mm sphere around the hand area (MNI coordinates: x = -32, y = -26, z = 58) ^13^, such that we obtained FA, NDI, ODI and IVF values solely within the part of the hand area sphere that overlapped with the individual cortical ribbon and left M1 mask simultaneously. The sphere was created using MarsBaR implemented in SPM (<http://marsbar.sourceforge.net/>). Supplementary Figure 3 shows the correlation coefficients between FA and NODDI values the hand area GM mask, which are very similar to the main analysis that focused on the whole left M1 GM mask (Supplementary Figure 1).

*
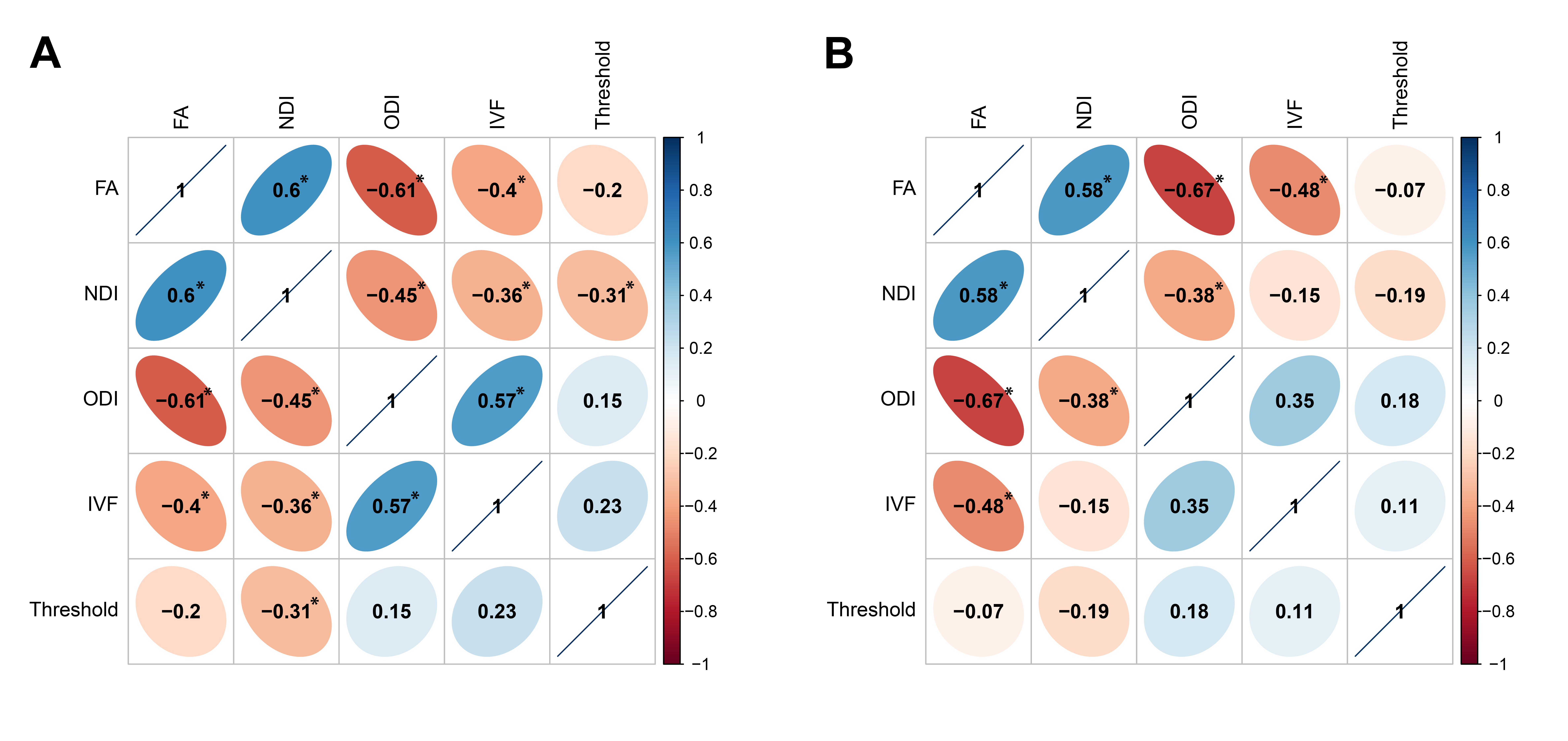
*

Supplementary Figure 3. Correlation coefficients of the correlation between FA, NDI, ODI, IVF within the hand area and motor threshold in (A) MS and (B) HC. * *p* < .05 (FDR corrected). Abbreviations: GM, grey matter, FA, fractional anisotropy, NDI, neurite density index, ODI, orientation dispersion index, IVF, isotropic volume fraction, MS, multiple sclerosis, HC, healthy controls.

*S9. Correlation analysis of FA and NODDI measures within the left M1 mask of the SMATT and motor threshold*

FA, NDI, ODI and IVF values within the white matter were extracted by overlaying the tract that originates in left M1 of the SMATT ^14^ on the diffusion maps and correlated with each other, as well as with the resting motor threshold. Average FA and NODDI values within the white matter tract originating in left M1 did not correlate with motor threshold (Supplementary Figure 4).


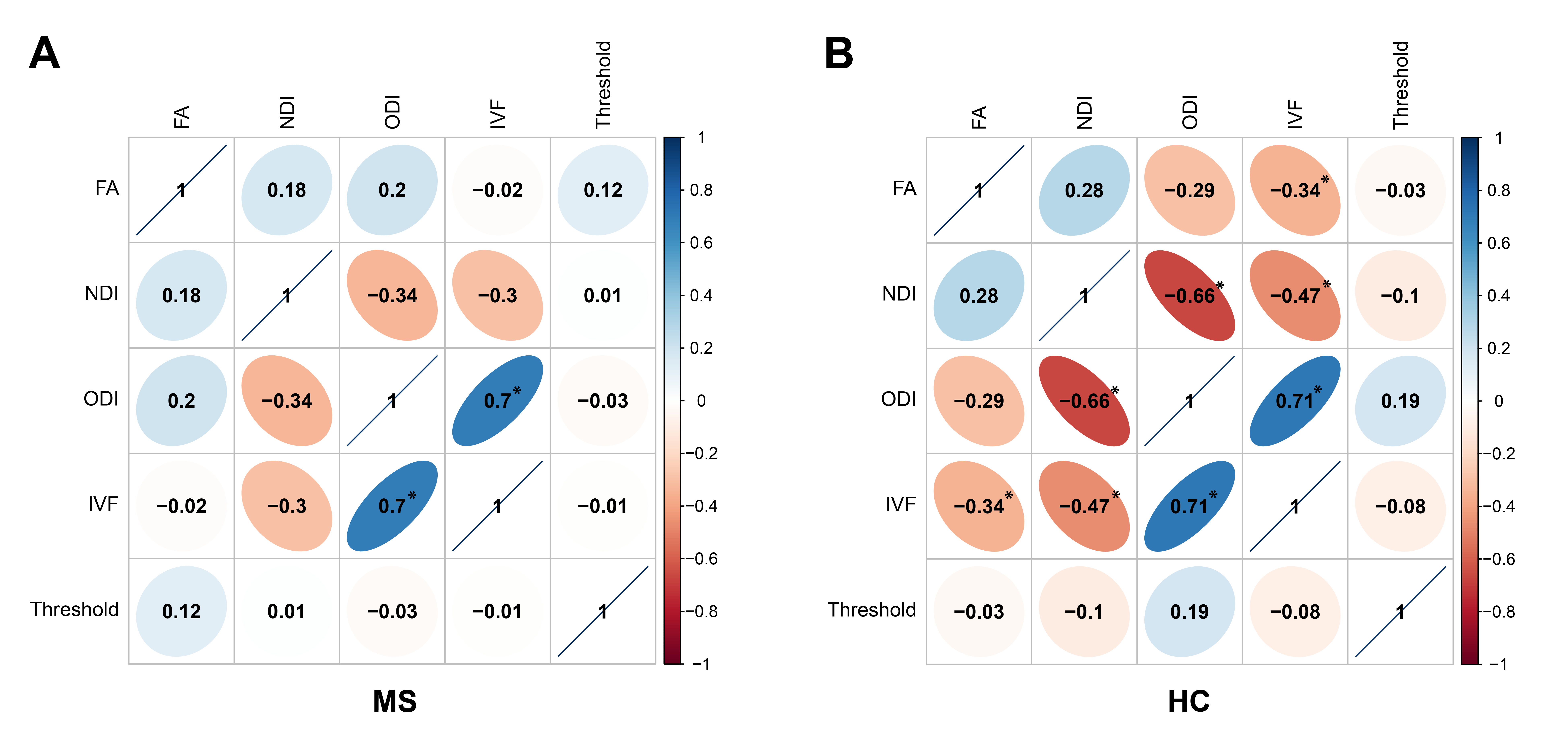


Supplementary Figure 4. Correlation coefficients of the correlation between FA, NDI, ODI, IVF within the left M1 mask of the SMATT and motor threshold in (A) MS and (B) HC. * *p* < .05 (FDR corrected). Abbreviations: GM, grey matter, FA, fractional anisotropy, NDI, neurite density index, ODI, orientation dispersion index, IVF, isotropic volume fraction, MS, multiple sclerosis, HC, healthy controls, SMATT, sensorimotor area tract template.

*S10. TBSS analysis applied to JHU atlas*

We additionally provide results of a congruent analysis regarding Johns Hopkins University (JHU) DTI-based WM atlas that also covers tracts outside the motor system ^10–12^.

We computed the number and percentage of voxels showing group differences for each region of the JHU atlas for FA, NDI and ODI maps (Supplementary Table 7). Fibers exhibiting group differences in a large part of the region in either FA, NDI or ODI or combinations of them included fornix, thalamic radiation, internal and external capsule, corpus callosum, corona radiata and sagittal stratum.

Supplementary Table 9. Number and percentage of significant voxels in JHU regions for FA, NDI and ODI. The last column contains the total number of voxels of the mean skeleton in each region. Contrast HC > MS for FA and NDI, and MS > HC for ODI, sorted decreasingly by the percentage of voxels showing significant group differences of each region, summed over FA, NDI and ODI

| **JHU label** | **FA (%)** | **NDI (%)** | **ODI (%)** | **Total** |
| --- | --- | --- | --- | --- |
| Left fornix (cres) / stria terminalis | 323 (28.7) | 268 (23.8) | 297 (26.4) | 1125 |
| Right fornix (cres) / stria terminalis | 296 (26.3) | 187 (16.6) | 294 (26.4) | 1124 |
| Left posterior thalamic radiation | 1128 (28.4) | 1144 (28.8) | 401 (10.1) | 3978 |
| Left retrolenticular part of internal capsule | 614 (24.9) | 668 (27.1) | 224 (9.1) | 2469 |
| Right posterior thalamic radiation | 1136 (28.6) | 1168 (29.4) |  | 3972 |
| Body of corpus callosum | 3076 (22.3) | 3003 (21.9) | 1666 (12.2) | 13711 |
| Right sagittal stratum | 530 (23.8) | 586 (26.3) |  | 2228 |
| Genu of corpus callosum | 1734 (19.6) | 1733 (19.6) | 860 (9.7) | 8851 |
| Left sagittal stratum | 450 (10.2) | 476 (21.3) | 89 (4) | 2231 |
| Left external capsule | 879 (15.7) | 805 (14.4) | 775 (13.9) | 5587 |
| Left anterior corona radiata | 1145 (16.7) | 1505 (22) | 138 (2) | 6852 |
| Right retrolenticular part of internal capsule | 445 (17.7) | 577 (22.9) | 1 (0.04) | 2515 |
| Right posterior corona radiata | 690 (18.5) | 759 (20.4) | 20 (0.5) | 3728 |
| Splenium of corpus callosum | 2295 (18) | 2359 (18.5) | 120 (0.9) | 12729 |
| Left superior longitudinal fasciculus | 968 (14.7) | 1402 (21.2) | 3 (0.04) | 6605 |
| Fornix (column and body) | 80 (12.1) | 70 (10.6) | 86 (13.1) | 659 |
| Right anterior corona radiata | 714 (10.4) | 1449 (21.2) | 251 (3.7) | 6849 |
| Right superior longitudinal fasciculus | 874 (13.2) | 1411 (21.4) |  | 6607 |
| Left posterior corona radiata | 573 (15.4) | 684 (18.4) | 21 (0.6) | 3714 |
| Left superior corona radiata | 901 (12) | 1226 (16.3) | 202 (2.7) | 7508 |
| Right superior corona radiata | 775 (10.3) | 1345 (17.9) | 198 (2.6) | 7500 |
| Right cerebral peduncle | 359 (15.8) |  | 282 (12.4) | 2278 |
| Left superior fronto-occipital fasciculus | 3 (0.6) | 106 (20.9) | 29 (5.7) | 507 |
| Right anterior limb of internal capsule | 216 (6.9) | 88 (2.8) | 532 (17) | 3138 |
| Right superior fronto-occipital fasciculus |  | 73 (14.4) | 45 (8.9) | 507 |
| Right cingulum (hippocampus) | 103 (8) | 175 (14.2) |  | 1236 |
| Left posterior limb of internal capsule | 12 (0.3) | 407 (10.8) | 373 (9.9) | 3752 |
| Left uncinate fasciculus | 33 (8.8) | 22 (2.9) | 23 (6.1) | 376 |
| Right cingulum (cingulate gyrus) | 120 (5.1) | 323 (13.8) |  | 2342 |
| Left cerebral peduncle |  | 5 (0.2) | 421 (18.5) | 2278 |
| Left anterior limb of internal capsule | 2 (0.1) | 95 (3.1) | 438 (14.5) | 3018 |
| Left cingulum (hippocampus) |  | 200 (17.3) |  | 1155 |
| Right external capsule | 378 (6.7) | 558 (9.9) |  | 5611 |
| Left cingulum (cingulate gyrus) | 42 (1.5) | 390 (14.2) |  | 2751 |
| Right posterior limb of internal capsule | 307 (8.2) | 89 (2.4) | 160 (4.3) | 3754 |
| Right tapetum | 36 (6) | 36 (6) |  | 596 |
| Right corticospinal tract | 29 (2.1) |  | 133 (9.8) | 1362 |
| Right superior cerebellar peduncle |  |  | 81 (8.2) | 992 |
| Right inferior cerebellar peduncle |  |  | 79 (8.2) | 968 |
| Right medial lemniscus |  |  | 28 (4.1) | 690 |
| Middle cerebellar peduncle |  |  | 595 (3.8) | 15644 |
| Right uncinate fasciculus | 2 (0.5) | 8 (2.1) |  | 380 |
| Left tapetum | 4 (0.7) | 4 (0.7) |  | 600 |
| Left corticospinal tract |  |  | 9 (0.7) | 1370 |
| Pontine crossing tract |  |  | 7 (0.5) | 1500 |
|  |  |  |  |  |
| **TOTAL** | **21272 (12.7)** | **25404 (15.2)** | **8881 (5.3)** | **167347** |

*Note:* Abbreviations: FA, fractional anisotropy, NDI, neurite density index, ODI, orientation dispersion index, HC, healthy control, MS, multiple sclerosis.

Again, we disentangled if one of the parameters alone or if combinations of FA, NDI and ODI contributed to observed group differences. The percentage of all single parameters and their combinations are depicted for each JHU region is presented in Supplementary Figure 5A, and averages over all regions is depicted in Supplementary Figure 5B. Neurite density exclusively (49%) and in combination with FA (29%) accounted for the largest amount of differences, followed by a combination of NDI, ODI and FA (9%) and ODI alone (6%).


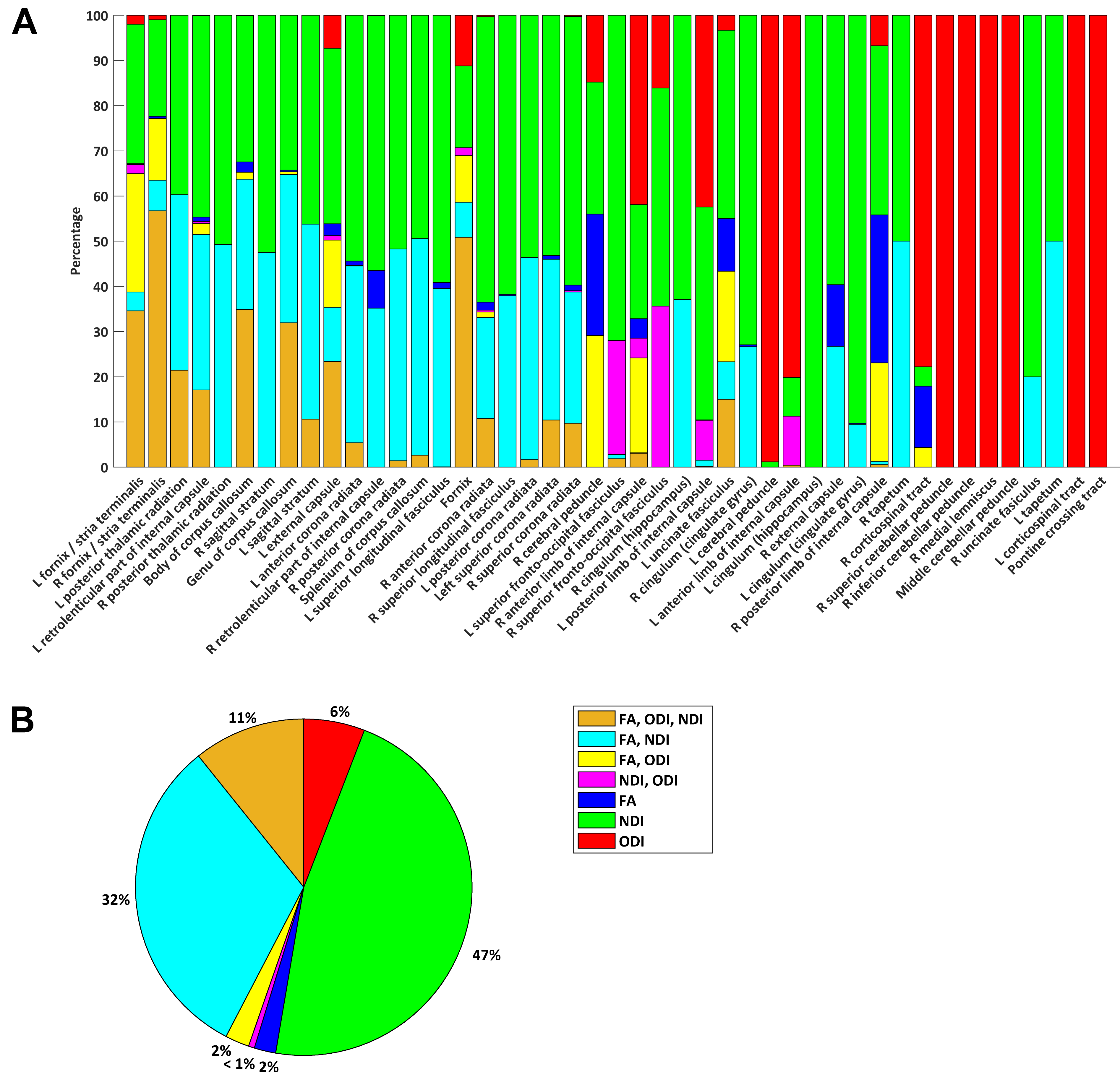


Supplementary Figure 5. Percentage of FA, NDI and ODI separately or combined showing group differences in the tract skeleton in intersection with JHU regions (A). Percentage averaged over regions with any significant group difference (B). Abbreviations: L, left, R, right, FA, fractional anisotropy, NDI, neurite density index, ODI, orientation dispersion index

**Supplementary** **References**

1. Caramia MD, Palmieri MG, Desiato MT, et al. Brain excitability changes in the relapsing and remitting phases of multiple sclerosis: a study with transcranial magnetic stimulation. Clin Neurophysiol. 2004;115:956–965.

2. Vucic S, Burke T, Lenton K, et al. Cortical dysfunction underlies disability in multiple sclerosis. Multiple Sclerosis Journal. 2012;18:425–432.

3. Boorman ED, O’Shea J, Sebastian C, Rushworth MFS, Johansen-Berg H. Individual Differences in White-Matter Microstructure Reflect Variation in Functional Connectivity during Choice. Current Biology [online serial]. 2007;17:1426–1431. Accessed at: http://www.sciencedirect.com/science/article/pii/S0960982207017204. Accessed July 10, 2020.

4. Calabrese M, Atzori M, Bernardi V, et al. Cortical atrophy is relevant in multiple sclerosis at clinical onset. Journal of Neurology. 2007;254:1212–1220.

5. Wahl M, Lauterbach-Soon B, Hattingen E, et al. Human Motor Corpus Callosum: Topography, Somatotopy, and Link between Microstructure and Function. J Neurosci [online serial]. Society for Neuroscience; 2007;27:12132–12138. Accessed at: https://www.jneurosci.org/content/27/45/12132. Accessed July 10, 2020.

6. Zipser CM, Premoli I, Belardinelli P, et al. Cortical excitability and interhemispheric connectivity in early relapsing–remitting multiple sclerosis studied with TMS-EEG. Frontiers in neuroscience. 2018;12:393.

7. De Santis S, Bastiani M, Droby A, et al. Characterizing microstructural tissue properties in multiple sclerosis with diffusion MRI at 7 T and 3 T: the impact of the experimental design. Neuroscience. Elsevier; 2019;403:17–26.

8. Spanò B, Giulietti G, Pisani V, et al. Disruption of neurite morphology parallels MS progression. Neurol Neuroimmunol Neuroinflamm. 2018;5:e502.

9. Zhang H, Schneider T, Wheeler-Kingshott CA, Alexander DC. NODDI: practical in vivo neurite orientation dispersion and density imaging of the human brain. NeuroImage. 2012;61:1000–1016.

10. Hua K, Zhang J, Wakana S, et al. Tract probability maps in stereotaxic spaces: analyses of white matter anatomy and tract-specific quantification. NeuroImage. 2008;39:336–347.

11. Mori S, Wakana S, Van Zijl PC, Nagae-Poetscher LM. MRI atlas of human white matter. Amsterdam: Elsevier Science; 2005.

12. Wakana S, Caprihan A, Panzenboeck MM, et al. Reproducibility of quantitative tractography methods applied to cerebral white matter. Neuroimage. 2007;36:630–644.

13. Rehme AK, Eickhoff SB, Rottschy C, Fink GR, Grefkes C. Activation likelihood estimation meta-analysis of motor-related neural activity after stroke. Neuroimage. 2012;59:2771–2782.

14. Archer DB, Vaillancourt DE, Coombes SA. A template and probabilistic atlas of the human sensorimotor tracts using diffusion MRI. Cerebral Cortex. Oxford University Press; 2018;28:1685–1699.
